# Estimating national dioxins and furans emissions, major sources, intake doses, and temporal trends in Iran from 1990–2010

**DOI:** 10.1186/s40201-017-0283-1

**Published:** 2017-10-10

**Authors:** Fatemeh Momeniha, Sasan Faridi, Heresh Amini, Mansour Shamsipour, Kazem Naddafi, Masud Yunesian, Sadegh Niazi, Kimiya Gohari, Farshad Farzadfar, Ramin Nabizadeh, Adel Mokammel, Amir Hossein Mahvi, Alireza Mesdaghinia, Homa Kashani, Simin Nasseri, Akbar Gholampour, Reza Saeedi, Mohammad Sadegh Hassanvand

**Affiliations:** 1 Center for Solid Waste Research (CSWR), Institute for Environmental Research (IER), Tehran University of Medical Sciences, Tehran, Iran; 2 Department of Environmental Health Engineering, School of Public Health, Iran University of Medical Sciences, Tehran, Iran; 3 Center for Air Pollution Research (CAPR), Institute for Environmental Research (IER), Tehran University of Medical Sciences, Tehran, Iran; 4 Department of Environmental Health Engineering, School of Public Health, Tehran University of Medical Sciences, Tehran, Iran; 5 Department of Epidemiology and Public Health, Swiss Tropical and Public Health Institute (Swiss TPH), University of Basel, Basel, Switzerland; 6 Department of Research Methodology and Data Analysis, Institute for Environmental Research, Tehran University of Medical Sciences, Tehran, Iran; 7 Department of Biostatistics, Faculty of Paramedical Sciences, Shahid Beheshti University of Medical Sciences, Tehran, Iran; 8 Non-Communicable Diseases Research Center, Endocrinology & Metabolism Population Sciences Institute, Tehran University of Medical Sciences, Tehran, Iran; 9 Department of Environmental Health Engineering, School of Health, Ardabil University of Medical Sciences, Ardabil, Iran; 10 Center for Water Quality Research (CWQR), Institute for Environmental Research (IER), Tehran University of Medical Sciences, Tehran, Iran; 11 Department of Environmental Health Engineering, School of Public Health, Tabriz University of Medical Sciences, Tabriz, Iran; 12 Department of Health Sciences, Faculty of Health, Safety and Environment, Shahid Beheshti University of Medical Sciences, Tehran, Iran

**Keywords:** Dioxins, Furans, UNEP toolkit, Intake dose, Iran, PCDD/PCDFs

## Abstract

**Background:**

Polychlorinated dibenzo-p-dioxins (PCDD) and dibenzofurans (PCDFs) are highly toxic persistent organic pollutants (POPs), which can cause various health outcomes, such as cancer. As a part of the National and Sub-national Burden of Disease Study (NASBOD), we aimed to estimate dioxins and furans national emissions, identify their main sources, estimate daily intake doses, and assess their trend from 1990–2010 in Iran.

**Methods:**

The Toolkit for Identification and Quantification of Releases of Dioxins, Furans and Other Unintentional POPs, which is developed by the United Nations Environment Programme (UNEP 2013), was used to estimate the emissions of PCDD/PCDFs from several sources into the air, water, land, residue, and other products. The daily intake doses were estimated using a linear regression of estimated emissions by UNEP Toolkit and average intake doses in other countries. Finally, the trend of PCDD/PCDFs emissions and daily intake doses were explored from 1990–2010.

**Results:**

The total emissions were estimated as 960 g Toxic Equivalents (g TEQ) for 1990 and 1957 g TEQ for 2010 (18.2 and 26.8 g TEQ per million capita, respectively). The estimations suggest that albeit contribution of open burning to PCDD/PCDFs emissions has been declining from 1990 to 2010, it remained the major source of emissions in Iran contributing to about 45.8% out of total emissions in 1990 to 35.7% in 2010. We further found that PCDD/PCDFs are mostly emitted into the ambient air, followed by residue, land, products, and water. The daily intake doses were estimated to be 3.1 and 5.4 pg TEQ/kg bw/day for 1990 and 2010, respectively. We estimated an increasing trend for PCDD/PCDFs emissions and intake doses in Iran from 1990–2010.

**Conclusions:**

The high levels of emissions, intake doses, and their increasing trend in Iran may pose a substantial health risk to the Iranian population. Further studies with more rigorous methods are recommended but this should not circumvent taking appropriate policy actions against these pollutants. Currently, Iran has no standard for dioxins and furans. Adaptation of the World Health Organization recommended guidelines might be an appropriate starting point to control dioxins and furans emissions.

## Background

Polychlorinated dibenzo-p-dioxins and polychlorinated dibenzofurans (PCDD/PCDFs) are unwanted environmental persistent organic pollutants (POPs) [1–3]. PCDD/PCDFs accumulate in the food, mainly in the fatty tissue of animals, hence are abundantly present in meats, dairies, and eggs [4]. The International Agency for Research on Cancer (IARC) has classified 2,3,7,8-tetrachlorodibenzo-p-dioxin, the most toxic dioxin, as carcinogenic to humans (IARC Group 1). [5]. According to studies, exposure to PCDD/PCDFs can cause a variety of adverse human health effects, such as reproductive and developmental disorders, detrimental impacts on immune systems, interference with hormones, poorer semen parameters, and occurrence of cancers [6–8]. Studies have also suggested that exposure to PCDD/PCDFs during the perinatal period may be associated with increased birth weight [9, 10]. The disability-adjusted life years due to PCDD/PCDFs in six selected European countries (Belgium, Finland, France, Germany, Italy, and the Netherlands) is estimated to range from 200 to 600 per million people [1]. Furthermore, there are some reports that a strong dose-response relationship between mortality and exposure to dioxins and furans is found [11, 12].

Despite of numerous negative consequences associated with exposure to PCDD/PCDFs [13, 14], only few studies have been conducted on emissions of PCDD/PCDFs in Iran [15, 16]. This study is the first to estimate the trend of emissions of PCDD/PCDFs across two decades in Iran. We borrowed information from other studies [17, 18], to estimate the intake doses at the national level. This study has been conducted as a part of the National and Sub-national Burden of Disease (NASBOD) study in Iran [19, 20].

We aimed to 1) estimate the national PCDD/PCDFs emissions, 2) estimate the intake doses of Iranian population, 3) identifying the main sources, and finally 4) assess trend of PCDD/PCDFs emissions and daily intake doses from 1990–2010 at national level.

## Methods

### Emission estimation of PCDD/PCDFs in Iran from 1990–2010

As the measurements of PCDD/PCDFs are complex, there is no reliable estimation of emissions in most of low- and middle-income countries. In order to help countries identify sources of emissions and to estimate release of PCDD/PCDFs, the United Nations Environment Program (UNEP) has established a Toolkit for Identification and Quantification of Releases of Dioxins, Furans and Other Unintentional Persistent Organic Pollutants (POPs) [21, 22]. The main objective of the Toolkit is to estimate the PCDD/PCDFs emissions from existing sources to the air, water, land, residue, and other products [22]. Therefore, emissions of PCDD/PCDFs were estimated by the methods proposed by the UNEP Toolkit, which consists of five-steps [21–23]. They are 1) the use of screening matrix to identify the major source categories of PCDD/PCDFs, 2) controlling subcategories to determine present activities and sources in the country, 3) collection of detailed information on the processes and classifying processes into similar groups using Standard Questionnaire, 4) quantifying identified sources with default/measured emission factors, and 5) establishment of full inventory, and reporting the results using standard guidelines [22].

Sources of PCDD/PCDFs emission in this Toolkit have been categorized into ten main groups. However, there are also some subgroups within each group. The ten main groups of PCDD/PCDFs emission are: 1) waste incineration (with seven subgroups), 2) ferrous and nonferrous metal production (with twelve subgroups), 3) heat and power generation (with five subgroups), 4) production of mineral products (with seven subgroups), 5) transportation (with seven subgroups), 6) open burning processes (with two subgroups), 7) production and use of chemicals and consumer goods (with eight subgroups) 8) disposal and landfill (with five subgroups), 9) contaminated sites and hotspots (with thirteen subgroups), and 10) miscellaneous activities (with five subgroups).

To achieve the aims of this study, we first identified the sources of PCDD/PCDFs emissions. Afterwards, we collected data from related organizations to estimate the levels of emissions using standard questionnaires developed by the UNEP. Finally, we estimated the levels of emissions for each source and for each year (starting from 1990 to 2010) using the emission factors determined in the Toolkit. [15, 16, 24]. We used the following equation to estimate PCDD/PCDFs emissions released per year:

PCDD/PCDFs emission per year = Emission factor_(Air, Water, Land, Product, Residue)_ × Activity Rate.

The emission of PCDD/PCDFs per year is given in grams Toxic Equivalents (TEQ) per year. Activity rate is the value of feed material processed or product produced in litters or tonnes per year.

### Estimation of intake doses in Iran from 1990–2010

Daily intake doses of PCDD/PCDFs were estimated using the equation from regression analysis between per capita emissions of PCDD/PCDFs that was estimated based on the UNEP Toolkit, and intake doses in some European countries including Germany, England, Norway, and Finland. Countries were selected based on their concurrent availability of data on PCDD/PCDFs emission levels and intake doses. [25, 26]. Linear regression analysis between PCDD/PCDFs emission per capita and intake dose levels of PCDD/PCDFs (pg TEQ/kg/d) were calculated (Table 1). Finally, the eq. Y = 0.2484X − 1.3525 was reached to estimate the intake doses according to PCDD/PCDFs emissions. Where, Y is the daily intake dose of PCDD/PCDFs (pg TEQ/kg/d) and X is estimated annual emission of PCDD/PCDFs per capita according to UNEP Toolkit. The adjusted R^2^ and RMSE (root-mean-square error) were 0.86 and 0.45, respectively. Using the above mentioned equation, we also estimated the intake doses of PCDD/PCDFs from 1990 to 2010 (Table 2).

**Table 1:** Data used for estimating intake doses of PCDD/PCDFs

Country	Annual Estimated PCDD/PCDFs Emission (g TEQ/year)	Annual Estimated PCDD/PCDFs Emission per million capita (g TEQ)	Intake dose	
			(pg TEQ/day)	(pg TEQ/kg/day)
Germany [16]	840	10.22	65.10	1.09
United Kingdom [17]	930	15.80	175.50	2.93
Norway [18]	40	8.90	65.00	1.08
Finland [19]	70	13.73	95.00	1.58

**Table 2:** PCDD/PCDFs emission per capita (million) in Iran and other countries in 2010

Region	Emission per capita (g TEQ/million)	References
Iran	26.6	Current study
France	18.7	(Momeniha et al., [15])
Italy	19.1	
UK	15.8	
Germany	10.2	
Belgium	48.0	
Spain	8.1	
Switzerland	25.0	
Portugal	13.0	
Austria	14.8	
Greece	11.3	
Netherlands	7.5	
Sweden	10.1	
Finland	13.7	
Jordan	10.0	
Denmark	9.4	
Luxemburg	125.0	
Norway	8.9	
Lebanon	13.0	
Ireland	9.2	
Haifa district	1.8	
Turkey	13.3	(Saral et al., [28])
Argentina	23.4	
Australia	25.1	
Ecuador Republic	4.8	
Estonia	10.0	
Philippines	3.9	
Croatia	25.8	
Cambodia	20.4	
Cuba	17.4	
Latvia	6.5	
Lithuania	14.1	
Paraguay	13.6	
Poland	12.7	
Sri Lanka	8.6	
Chile	3.3	
Thailand	4.6	
Uruguay	5.7	
Jordan	12.1	
Vietnam	0.2	
Zambia	28.2	
Cyprus	7.5	
Czech Republic	31.1	
Estonia	6.2	
Malta	9.8	
Romania	22.5	
Bulgaria	38.5	
Slovakia	33.0	
Hungary	12.1	
Taiwan	2.9	
Slovenia	18.0	

## Results and discussion

### Estimated PCDD/PCDFs emissions in Iran from 1990–2010

**Fig. 1:**
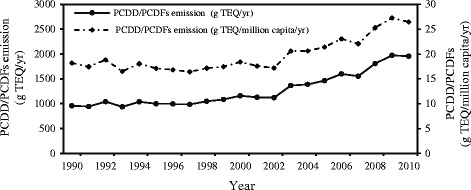
Estimated PCDD/PCDFs emissions in Iran from 1990 to 2010

The PCDD/PCDFs emission levels were estimated to be about 960 g TEQ in 1990 and 1960 g TEQ in 2010 (Fig. 1). We estimated that there has been an increase of about 100% in emissions of PCDD/PCDFs over a 20-year period from 1990 to 2010. The rate of increase has been particularly dramatic from 2000 to 2010. The level of PCDD/PCDFs emissions in the Central, East and Northeast provinces of Iran is greater than that of the Southern, Western, Northern, and Northwestern provinces (unpublished results). This might be due to the extensive growth in the production of industrial processes, and increased transportation fleet. Conversely, some developed countries, such as England have experienced a steady decline in emission of these pollutants since 1990 through implementation of emission control regulations [27].

Figure 1 shows the time trends of PCDD/PCDFs emission per capita in Iran from 1990–2010. There has been an increase in the levels of PCDD/PCDFs emissions per capita during this period. Moreover, as demonstrated, the PCDD/PCDFs emission per millions of people in Iran to other countries ranged from 0.21 to 14.69, with Haifa district recording the highest value. This ratio has been less than unity for Belgium, Bulgaria, and Czech Republic. Also, the PCDD/PCDFs emission per millions for Turkey, Chile, Philippines, and Thailand has been 10.8, 8.02, 6.82, and 5.78, respectively [28].

### Key sources of PCDD/PCDFs emissions in Iran from 1990–2010

**Fig. 2:**
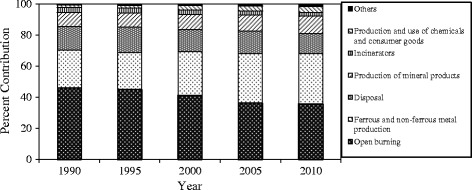
Estimated contribution of the key emission sources to PCDD/PCDFs in Iran from 1990 to 2010

As shown in Fig. 2, open burning, ferrous and non-ferrous metal production, disposal, production of mineral products, and waste incinerator accounted for 94.4% to 97.6% of the total emissions of PCDD/PCDFs in Iran. Although, our results showed that the contribution of open burning to PCDD/PCDFs emission has been declining from 1990 to 2010, it remains the main source of PCDD/PCDFs emissions in Iran contributing to about 45.8% in 1990 to 35.7% in 2010 out of total emissions. This decline might be due to some regulations banning open burning in all sectors of the economy. Nonetheless, the contribution of ferrous and non-ferrous metal production to the total emissions of PCDD/PCDFs at the national level has been increasing. This can be attributed to the development in industries requiring chemical processes.

**Fig. 3:**
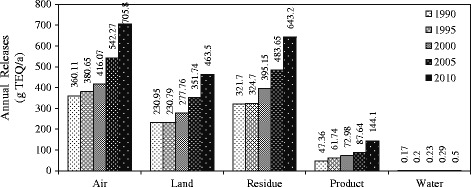
Estimated annual PCDD/PCDFs releases into the environment matrix

Figure 3 shows PCDD/PCDFs released into all environmental matrixes. It indicates that emissions of these chemicals into the air is predominant, followed by residue, land, products and water. It also presents the increase of the total emission of PCDD/PCDFs into all matrixes over the years.

### PCDD/PCDFs intake doses in Iran from 1990–2010

**Fig. 4:**
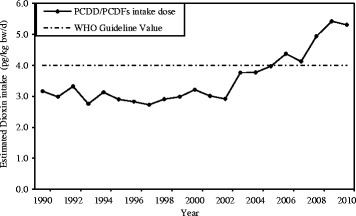
Estimated PCDD/PCDFs intake doses at national level from 1990 to 2010. The dash lines indicate WHO guideline level (4 pg TEQ/kg bw/d)

The national daily intake level of PCDD/PCDFs ranged from 3.1 pg TEQ/kg/d to 5.4 pg TEQ/kg/d, from 1990 to 2010 (Fig. 4). Findings indicate on average, an increase of about 8.7% each year from 1990 to 2010. Although, the WHO has recommended 4 pg TEQ/kg/d as the standards for PCDD/PCDFs, generally known as tolerable daily intakes (TDIs), numerous regulatory agencies in each country have their own TDIs. The proposed TDIs for PCDD/PCDFs ranges from 1 pg TEQ/kg/d (in the Netherlands and Germany), to 4 pg TEQ/kg/d (in Japan), 5 pg TEQ/kg/d (in Sweden, Norway, Finland and Denmark), and to 10 pg TEQ/kg/d (in UK, New Zealand and Canada) [29, 30]. Currently, Iran has no PCDD/PCDFs standard value. Adapting the World Health Organization recommended guideline value might be a starting point against these pollutants in Iran.

**Fig. 5:**
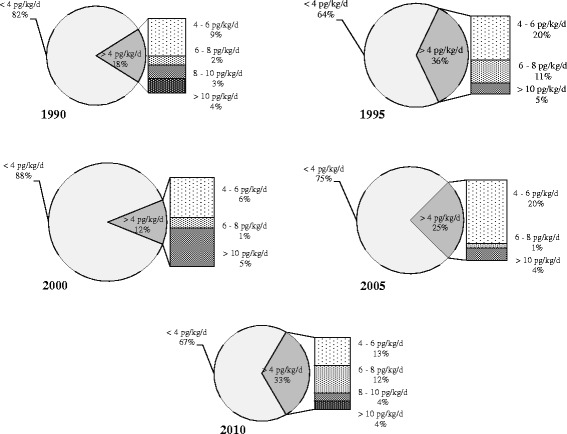
The percentage of national intake doses of PCDD/PCDFs in Iran from 1990 to 2010. The cut off of 4 pg/kg/d is the WHO recommended guideline value

As illustrated in Fig. 5, approximately 12–36% of exposure to PCDD/PCDFs (within several countries including Iran) exceeds the daily intake level recommended by the WHO.

This work has several limitations due to the large number of data used in estimating PCDD/PCDFs. Besides, the emission factors in the UNEP Toolkit used to estimate PCDD/PCDFs may be different for each country. There are only few published data on PCDD/PCDFs emissions in Iran and our estimations might be over/under-estimation of real emissions and intake doses. There were also limited data from other countries, which may affect the regression analysis of the daily intake level of PCDD/PCDFs.

## Conclusions

This study is first to estimate spatial and temporal trend of PCDD/PCDFs emission and its intake doses in Iran from 1990 to 2010. We estimated PCDD/PCDFs emissions from different sources, such as open burning processes, ferrous and nonferrous metal production, disposal, production of mineral products, waste incineration, chemicals production and consumer goods use and other sources. Our analyses showed that open burning and ferrous/nonferrous metal production contributed significantly to PCDD/PCDFs emissions in Iran. In fact, open burning processes and production of ferrous/nonferrous metal, respectively, in 1990 to 2010 contribute to 45.8% to 35.7% and 24.4% to 32.2% of the total emissions into the environment. The results indicate a significant growth in the emission level of PCDD/PCDFs over a 20-year period from 1990 to 2010. The growth in the level of emission can be, however, attributed to the sudden increase in the proportion of people living in the urban areas, and the growth in industries across the country. The intake level of PCDD/PCDFs emissions at national scale has also increased by 70% from 1990 to 2010. Approximately 12–36% of exposure to PCDD/PCDFs exceeds the daily intake level recommended by the WHO. Our data further revealed a higher per capita (in million) emission of PCDD/PCDFs in Iran in comparison with other Asian countries. Besides, the daily intakes of PCDD/PCDFs from 2006 to 2010 exceed the WHO’s guideline of 4 pg TEQ/kg bw/d. Considering the adverse effects of these risk factors on human health and the environment, results of this study suggest that appropriate policy actions are required by Iran to control emission of PCDD/PCDFs and its associated risk factors.

## Abbreviations

IARC: International Agency for Research on Cancer
NASBOD: National and Sub-national Burden of Disease
PCDD/PCDFs: Polychlorinated dibenzo-p-dioxins and polychlorinated dibenzofurans
POPs: Persistent Organic Pollutants
RMSE: Root-Mean-Square Error
TCDD: 2,3,7,8-Tetrachlorodibenzo-p-dioxin
TDIs: Tolerable Daily Intakes
TEQ: Toxic Equivalents
UNEP: United Nations Environment Program
WHO: World Health Organization
